# Nurses’ Perceptions of Spirituality and Spiritual Care and Influencing Factors in Türkiye, Italy, and Albania: A Multicultural Study

**DOI:** 10.3390/healthcare12141391

**Published:** 2024-07-11

**Authors:** Ebru Baysal, Hacer Demirkol, Ahmet Erol, Elif Deniz Kaçmaz, Blerina Duka, Benarda Agolli, Alessandro Stievano, Ippolito Notarnicola

**Affiliations:** 1 Department of Fundamentals Nursing, Faculty of Health Sciences, Manisa Celal Bayar University, 45030 Manisa, Türkiye; e_bay100@hotmail.com; 2 Department of Psychiatric and Mental Health Nursing, Faculty of Health Sciences, Yozgat Bozok University, 66000 Yozgat, Türkiye; 3 Department of Fundamentals Nursing, Faculty of Health Sciences, Batman University, 72040 Batman, Türkiye; ahmet.erol630@gmail.com; 4 Department of Psychiatric Nursing, Faculty of Health Sciences, Izmir Bakırcay University, 35665 Izmir, Türkiye; deniz.kacmaz@bakircay.edu.tr; 5 Department of Biomedicine and Prevention, University of Rome Tor Vergata, 00133 Rome, Italy; bleriduka@yahoo.it; 6 Maliq Primary Health Center, 7005 Korca, Albania; agollibenarda@gmail.com; 7 Department of Clinical and Experimental Medicine, University of Messina, 98100 Messina, Italy; alessandro.stievano@gmail.com; 8 Centre of Excellence for Nursing Scholarship OPI, 00136 Rome, Italy; ippo66@live.com

**Keywords:** spirituality, spiritual care, nursing, culture

## Abstract

The present study aimed to explore the perceptions of spirituality and spiritual care among nurses who work in three different countries with different cultures as well as the factors influencing their perception. This is a cross-sectional multicultural study conducted with a total of 1090 nurses from Türkiye, Albania, and Italy. Data were collected through the “Individual Information Form” and the “Spirituality and Spiritual Care Rating Scale”. Nurses’ mean score on the Spirituality and Spiritual Care Rating Scale was above average. It was also found that country, marital status, religious belief, Islamic religion, training for spiritual care, and using spiritual practices to cope with difficulties or illnesses influenced nurses’ perception of spirituality and spiritual care. It was concluded that nurses from the three countries associated spirituality and spiritual care with religion and that some personal characteristics influenced their perceptions of spiritual care. The results highlight the need for training in spiritual care to improve nurses’ perceptions and practices of spiritual care.

## 1. Introduction

Spirituality is an abstract and subjective concept with different definitions. For some, this idea represents a belief in a higher power, but for others, it refers to the search of meaning and purpose in life [1]. As a concept, spiritual care is defined as “the care confirming the unique value of the individuals based on unconditional love and being under the effect of their spiritual and cultural beliefs, physical conditions, emotions, thoughts, and cultural connections” [2]. In recent years, spiritual care has played a significant role in the care and treatment of patients. Besides their physical health problems, many patients face various emotional and social problems such as hopelessness, anxiety, anger, fear, stress, loneliness, loss of meaning in life, spiritual pain, and restlessness due to being unable to perform spiritual practices in the hospital setting [3]. All these complicated problems could lead to deterioration of individuals’ overall health by damaging their spiritual well-being. Hence, nurses should perform spiritual care as a part of holistic healthcare, address individuals’ spiritual and religious needs, and try to enhance emotional and spiritual well-being as well as physical well-being [4,5]. 

Many studies conducted with different patient populations in the literature reveal that spiritual care yields positive outcomes on physical symptoms such as fatigue and pain [6,7]; mental symptoms like depression, anxiety, and hopelessness [8]; and stress [7]. Spiritual care also positively contributes to improving patients’ quality of life, coping capacity, psychological resilience, and self-esteem, accelerating the recovery process, decreasing hospitalization time, and reducing hospital costs [4,5,9,10,11,12].

Spiritual care requires nurses to have high awareness and competency to perform effectively. However, many studies in the literature report low levels of nurse competency in spiritual care [13,14] and negligence of patients’ spiritual needs by nurses [15,16]. In addition, nurses face challenges integrating spiritual care into general clinical practices [17]. 

Spiritual care encompasses a wide range of religious and psychological aspects and moral and cultural bases [18]. Thus, nurses’ cultural and religious values are considered to influence their perceptions and practices related to spiritual care. In their study conducted in 2020 with Turkish nurses, Aslan and colleagues found that nurses’ awareness of spiritual care was insufficient, and they tended to see spirituality as a religious necessity [7]. Another analysis carried out in the United States of America reported that nurses did not feel ready to offer spiritual care [19]. In an examination performed in the Netherlands, it was contended that hospital nurses were competent in providing spiritual care and reported performing it monthly [20]. Still another investigation in South Korea found that most palliative care nurses had difficulty providing spiritual care [21]. In addition, professional/technical factors such as insufficient training, lack of time, educational background, clinical experience, and individual characteristics, as well as attitudes like the fear of religious conversion, fear of imposing their own beliefs, fear of offending the patient, confusion about the limits of spiritual care, and attitude towards death, which can be shaped by cultural and religious factors, have been reported to have a possible effect on spiritual care practice [5,9,15,16,21]. Considering all this evidence, it can be argued that nurses from different cultural and religious backgrounds have different perceptions and competency levels regarding spiritual care. Therefore, the present study aimed to explore spiritual care perceptions of nurses working in countries with different cultural characteristics, namely, Türkiye, Albania, and Italy, as well as the factors influencing their perception, and to contribute global data in this context into the literature.

## 2. Materials and Methods

### 2.1. Aim

The study aimed to determine the perceptions of spirituality and spiritual care among nurses working in three different countries and the factors that influence this perception. 

### 2.2. Design

The study was designed as an online cross-sectional multicultural study following the guidelines of the Strengthening Reporting of Observational Studies in Epidemiology (STROBE).

### 2.3. Setting and Sample

The study was led by nurses working in Türkiye, Albania, and Italy from July 2022 to July 2023. Nurses, who were over 18, had been working as a nurse at the outpatient or inpatient treatment units for at least three months, and who volunteered to participate in the research were included in the analysis. Snowball sampling, which is one of the non-probability sampling methods, was used to reach as many nurses as possible across three countries, as well as to contact nurses interested in spiritual care while saving time and effort [22]. To ensure as much homogeneity as possible in snowball sampling, a sample of nurses with a diverse range of characteristics (varying socioeconomic status, living in different regions, etc.) was initially reached. These people were also asked to share the online question form with their acquaintances. Thus, it was aimed to prevent the possibility of participants suggesting candidates with similar characteristics [23]. The sample size was estimated using the Global Health Workforce Statistics database in Health Workforce: Nursing Staff (2021) as a reference [24]. Nursing personnel numbers in Türkiye, Albania, and Italy are 227,292, 15,692, and 370,887, respectively. Using the Epi info 2000 statistical program (version 3.01; OpenEpi, Centers for Disease Control and Prevention (CDC), Atlanta, GA, USA), the minimum sample size for the study was calculated to be at least 384 nurses with a 95% confidence level and 5% variance. A convenience sample of 1974 registered nurses were invited to participate in the study. A total of 1138 nurses responded to the question form, and the investigation was completed with 1090 nurses after excluding the questionnaires with missing data.

### 2.4. Data Collection

The online question form developed over Google surveys by the researchers was shared with the nurses through social media (WhatsApp, Instagram, LinkedIn, Researchgate, etc.) and email. In the first section of the inquiry form, information was provided concerning the aim of the research, and written consent was obtained from the participants. Later, they were asked to fill in the online questionnaires, which took approximately 15 min to complete. The data were simultaneously collected upon receiving ethical committee approvals from each individual country. The questionnaire remained open for 12 months (from July 2022 to July 2023) in each country.

### 2.5. Instruments

The online questionnaire consists of two sections. The first section was the “Individual Information Form”, including the nurses’ socio-demographic characteristics and perceptions of spirituality and spiritual care. The form, which was developed by the researchers based on the related literature, consisted of 28 questions about the nurses’ attitudes and perceptions towards spiritual care practices and information such as their age, gender, professional experience, and unit of service. The individual information form was translated into Turkish, Albanian, Italian, and English by the researchers. Expert opinion was obtained in all languages, and necessary changes were accomplished accordingly. 

The second section of the questionnaire is the “Spirituality and Spiritual Care Rating Scale” (SSCRS). The scale was developed by McSherry et al. [25], and its validity and reliability were confirmed in Turkish [26]. Before collecting data from the nurses in Albania and Italy, the researchers examined the cultural validity and reliability of the SSCRS. Scales were used in the native language of each country, and written permission was obtained from the researchers who conducted validity–reliability studies of the scales. We used the guideline were originally developed with support from the American Academy of Orthopedic Surgeons (AAOS) for the process of cross-cultural adaptation of the scale for Italy and Albania [27]. First, the scale was translated into Italian and Albanian languages. Later, following the synthesis, back translation, expert committee review, and pretesting stages, it was decided that it was appropriate to use the scales. Cronbach alpha values were found to be 0.80 for Italy and 0.77 for Albania.

The SSCRS measures spirituality by quantifying participants’ perceptions of the extent to which they hold particular spiritual views and engage in certain spiritually related activities. The tool is composed of 17 items rated on a five-point Likert-type scale. The items are scored from 1, indicating “strongly disagree”, to 5, indicating “strongly agree”. The total item score approaching 5 indicates high perception levels of spirituality and spiritual care. Cronbach’s alpha coefficient of the SSCRS were as follows: Türkiye 0.75, Italy 0.79, and Albania 0.83 for the present study.

### 2.6. Data Analysis

The data were evaluated on the SPSS 21.0 software using descriptive statistical methods (numbers, percentages, means, standard deviation, medians). Skewness and kurtosis tests were performed to check the normality of data distribution, indicating that the groups were homogeneous. The characteristics of nurses’ perceptions of spirituality and spiritual care were compared among countries by χ^2^ and Fisher’s exact tests. The characteristics of the nurses and SSCRS scores were compared with the independent *t*-test, one-way analysis of variance (ANOVA), and Tukey’s HSD test. Multiple linear regression was used to identify the impacts of variables on their perceptions of spirituality and spiritual care. Significance was accepted as *p* < 0.05.

### 2.7. Ethical Considerations

This study was approved by the respective countries’ institutional ethics committees (Türkiye: 2022-33/24, Albania: 2022-PROT.069-07; Italy: 2022-21/2) and included nurses who voluntarily agreed to participate. The questionnaire forms were developed to be sent to nurses via doc.google.com URL to ensure data confidentiality. Before starting the questionnaire form, the participants were requested to read and approve the informed consent form explaining the purpose of the study. The questionnaire contained no questions about the participants’ contact or personal information. There was no financial inducement for people to participate in the investigation. The investigation was carried out following the ethical criteria of the Helsinki Declaration. 

## 3. Results

The nurses’ mean age was 37.50 ± 10.78 years, and mean professional experience was 13.13 ± 10.63 years. Most of the nurses were female (73%) and had a bachelor’s degree (73.8%), and 63.9% of them were married or had a partner. Approximately half (58.8%) worked in inpatient clinics while most of them were staff nurses (78.9%). A majority (87.7%) contended that they chose the profession willingly, and more than half (57.9%) had job satisfaction. Almost all nurses (93.2%) held a religious belief, and more than half (59.9%) embraced Islam. Less than half of the nurses (44.6%) reported performing religious practice regularly. The most frequent practices were meditation/namaz (prayer) (26.9%) and praying (25.7%). Most nurses (86.5%) stated that spiritual care practices were not performed at the hospital where they were working. A frequently utilized spiritual care practice is emotional support (34.1%). The biggest barrier to spiritual care practices was found to be the fact that the health team did not value the nurses’ spiritual care (35%). Most nurses (85.5%) expressed that spiritual care practices were necessary. Only 17.1% of the participants stated they received education on spiritual care practices, and 22.3% believed they had sufficient knowledge. Approximately three-fourths (74.5%) of the nurses reported believing in the healing power of spiritual care practices, and 28.8% stated that they used spiritual practices to cope with difficulties and illnesses. Nurses’ socio-demographic characteristics and perceptions of spirituality and spiritual care were found to vary significantly by country (*p* < 0.01) (Table 1). 

The mean scores for the SSCRS are given in Table 2. The total mean SSCRS score of the nurses was determined to be above the average level (3.20 ± 0.62) and showed statistically significant differences across the countries (*p* < 0.01). The two highest mean scores for SSCRS were obtained for item 2: “I think that nurses can provide spiritual care by acting in a compassionate, concerned and positive manner while giving care” (3.64 ± 1.23), and item 4: “I believe spirituality involves only going to a place of worship (mosque/church)” (3.66 ± 1.30). The items with the lowest means were item 5: “I think that spirituality is not concerned with belief in God or a supreme power and worship” (2.63 ± 1.11), and item 15: “I think that spirituality involves personal friendships and relationships” (2.64 ± 1.11). All the items on the scale and the total score for the SSCRS (54.40 ± 10.66) were found to vary significantly by country (Table 2).

The relationships between the total SSCRS and independent variables were analyzed by one-way ANOVA and independent *t*-test (Table 3). Statistically significant differences were found between nurses’ perceptions of spirituality and spiritual care and their country, age, marital status, education, working unit, professional experience, religious belief, type of religious belief, regular religious practice, training for spiritual care, use of spiritual practices to cope with difficulties or illnesses, and belief that spiritual care practices have a healing effect (*p* < 0.001). The mean score of SSCRS was found to be high in nurses who were working in Türkiye, were in the 20–30 age group, were single, had a master’s or doctorate degree, were working in outpatient clinics, had professional experience of <5 years, held a religious belief, embraced Islamic faith, performed regular religious practices, received education on spiritual care practices, used spiritual practices to cope with difficulties or illnesses, and believed in the healing effect of spiritual care practices (*p* < 0.001) (Table 3).

We employed multiple linear regression analysis to determine the factors influencing nurses’ perceptions of spirituality and spiritual care. The results of the analysis showed that the combination of socio-demographic and spirituality-related variables was significantly related to nurses’ perceptions of spirituality and spiritual care (F = 20.243, *p* < 0.001). Also, country, marital status, having a religious belief, embracing the Islamic faith, receiving training for spiritual care, and using spiritual practices to cope with difficulties or illnesses were found to be influential factors, accounting for 17% (R^2^ = 0.171) of the variation in nurses’ perceptions of spirituality and spiritual care (Table 4).

## 4. Discussion

The spiritual approach in nursing care is inseparable from holistic nursing care [28]. Spirituality and spiritual care, two interconnected concepts, are influenced by the cultural characteristics of nurses [29]. Therefore, the present investigation explored the perceptions of spirituality and spiritual care among nurses working in Türkiye, Albania, and Italy with different cultural values and the factors affecting these perceptions. When the items of the scale evaluating the perceptions of spirituality and spiritual support of the participating nurses were examined (mean of SSCRS), no significant difference was detected among the countries only in the mean score of the item “I think that nurses can provide spiritual care by inviting a religious official to the hospital on patient’s demand”. Based on these findings, it could be contended that there is a need for religious officials regarding spiritual care in all three countries. The literature includes studies indicating that nurses feel incompetent in spiritual care and that they expect spiritual care to be provided by religious officials [30,31,32].

The second highest mean score on the SSCRS scale for nurses was found to be on the item “I think that nurses can provide spiritual care by acting in a compassionate, concerned and positive manner while giving care”. This finding can be associated with the fact that the spirituality and spiritual care perceptions of the nurses participating in the study were above average. Parallel with this result, Harrad and colleagues (2019) found that nurses were willing to listen to their patients, accompany them, instill confidence, and respect their religious and cultural beliefs when providing spiritual care [33]. Additionally, it is reported that when nurses provide patients with a gentle, compassionate, and affectionate environment that facilitates acceptance and hope, patients’ spiritual life improves [28,34,35]. 

The item with the highest mean score on the scale was “I think that spirituality involves only going to a place of worship (mosque/church)”, while the lowest mean score belonged to the item “I think that spirituality is not concerned with belief in God or a supreme power and worship”. These determinations indicate that nurses who participated in the study associated spiritual care with religion. Although one study can be found to reports that nurses can distinguish between the concepts of spirituality and religion in the literature [36], many studies have concluded that nurses generally associate spirituality with religion [37,38,39]. These results can also be associated with the fact that most of the nurses participating from Türkiye, Albania, and Italy hold religious beliefs. Perceptions of spirituality and spiritual care (mean of SSCRS total score) of the nurses who participated in the study were above moderate (mean 3.20 ± 0.62). Based on this conclusion, the participating nurses’ perception of spirituality and spiritual care can be defined as “less clear”, compared with other studies [37,40,41,42]. This indicates the necessity of providing nurses with knowledge and training regarding spirituality and spiritual care. 

When the participating nurses’ spirituality and spiritual care scores (mean of SSCRS total score) were evaluated in terms of certain variables, the group with the highest mean scores was found to be the nurses working in Türkiye. Recent studies conducted in Türkiye have reported that nurses have high perceptions of spirituality [43] and provide spiritual care at a moderate frequency [44]. There was no valid and reliable measurement tool for Italy and Albania during the time of the research, and hence no study results were identified on the perceptions and practices of nurses working in Italy and Albania regarding spirituality and spiritual care. When studies conducted in different countries using the same measurement tool are compared, nurses in Türkiye, Albania, and Italy had lower average spirituality and spiritual care scores than their colleagues in Saudi Arabia and Indonesia [29,45]. However, when compared to the outcomes of another study conducted in Ethiopia, it was determined that nurses in Türkiye had higher average spirituality and spiritual care scores [46]. According to the study results, despite not being at the desired level, Türkiye is seen to be the only country with spiritual support units and where patients receive most spiritual care in clinics. In Türkiye, especially as of 2015, spiritual support units have been established in hospitals in many provinces, and reflections on providing spiritual support for patients have been going on rapidly [47]. All these positive steps were considered to enhance Turkish nurses’ perceptions and practices regarding spirituality and spiritual care. However, there is a need to include spiritual care into undergraduate curricula, offer awareness training on spiritual care to nurses, and establish a more significant number of spiritual care units in hospitals to improve nurses’ perceptions and practices of spiritual care in Albania, Italy, and Türkiye as well [44,47,48,49]. 

In the literature, some studies report that older [50,51], married [50], and more experienced [29,46,52] nurses have higher perceptions of spirituality and spiritual care, while some studies report higher scores for younger [37,46], single, and less experienced nurses [37]. On the other hand, there are studies indicating that age [29], marital status [29,46,53], and professional experience [53] do not lead to any difference in the scores. When these results are considered as a whole, it can be emphasized that there is yet to be a definitive consensus on the impact of these variables on nurses’ perceptions of spirituality and spiritual care, and it is evident that nurses’ characteristics could influence the scores. 

When the nurses were assessed regarding educational background, it was found that nurses holding a master’s or doctorate degree have higher scores of spirituality and spiritual care perception. This determination is consistent with the literature, where many analyses report that nurses’ perceptions of spirituality and spiritual care increase as their level of education gets higher [37,46,50]. It is expected that nurses’ knowledge and problem-solving skills concerning spiritual care will be enhanced along with the increased level of education [46], which seems to cause a positive increase in nurses’ perceptions of spirituality and spiritual care.

The analysis, where Cooper and colleagues (2019) compiled nine studies conducted on spirituality and spiritual care among nurses between 2010 and 2018, found that perceptions of spirituality and spiritual care were higher among nurses working in palliative care, pediatric, and psychiatry units [54]. In contrast, the scores of nurses working in outpatient clinics were found to be higher in the present investigation. However, none of the studies included in the compilation evaluated nurses working in outpatient clinics. Furthermore, excessive workload and long hours of nurses working in inpatient units could have caused this result [55]. This finding suggests that for future studies, it might be beneficial to include nurses working in outpatient clinics alongside those working in inpatient units because spiritual care is a type of care that should be especially emphasized in all areas of the hospital [18]. 

Similar to many previous examinations [50,53,56], in the present study, perceptions of spirituality and spiritual care were found to be high among nurses who received training on spiritual care. A recent investigation by Yanti and colleagues (2022) has reported that spiritual care training enhances nurses’ perceptions of spirituality and spiritual care [57]. It is a well-known fact that nurses need training in spirituality and spiritual care [52]. It would be beneficial to integrate spirituality-related courses into undergraduate curricula and increase the number of training activities on spiritual care for nurses worldwide [57,58]. 

It was found in the present study that nurses who hold a religious belief and perform regular religious practices have higher perceptions of spirituality and spiritual care. Similarly, in the study conducted by Chew and colleagues (2016), the scores of nurses with a religious belief were found to be higher, while the frequency of religious practice was seen not to affect the score [59]. On the other hand, Wu and Lin (2011) reported that having a religious belief did not affect nurses’ perceptions of spirituality and spiritual care [56].

In our investigation, nurses who embraced the Islamic faith were found to have higher perceptions of spirituality and spiritual care. However, it was also observed that the outcomes were different from each other in Albania and Türkiye, two countries where Muslim nurses are in the majority. In addition, in Italy, where the majority is Christian, nurses’ scores were higher than those of the nurses in Albania. In the research conducted by Taylor and colleagues (2023) examining 16 international studies, it was determined that Muslim nurses provided the most spiritual care compared with any other religions and beliefs, but that the results obtained from countries where Muslim nurses were in the majority were not consistent with one another [16]. Bringing all these conclusions together, although religion emerges as a factor to be highlighted explicitly regarding the perception and practice of spiritual care, it is understood that it would be incorrect to approach religion by detaching it from its cultural context [16].

It was found in the study that nurses who use spirituality to cope with challenges and illnesses in their personal life and believe in the healing power of spirituality had higher perceptions of spirituality and spiritual care. Studies in the related literature report that nurses who define themselves as spiritual have higher perceptions of spirituality and spiritual care [59], nurses with a solid spiritual perspective find it worthwhile to integrate spiritual care into nursing practices [60] and they are more likely to engage in spiritual care practices [61]. Based on these findings, it stands beneficial to raise more awareness among nurses who do not hold a religious belief or do not believe in the effect of spirituality on health regarding the influence of religion and spirituality on patients’ health [62].

### Limitations

Our investigation has several limitations. Due to the cross-sectional research design used in this study, it is not possible to explain the causal relationship among variables. In addition, since the data were collected via Google Forms, it was not possible to reach nurses who did not use social media instruments. Moreover, not all the cities of the involved countries could be reached because of using snowball sampling as a convenience method. Furthermore, some of the factors that could affect perceptions of spirituality and spiritual care were approached in the investigation. However, there could be other personal and organizational variables that could affect this perception.

## 5. Conclusions and Recommendations

According to findings, nurses associate spirituality and spiritual care with religion. Most nurses do not use spiritual care practices for the patients in their unit. The most frequently used spiritual care practice by nurses is emotional support. A significant barrier to spiritual care practices is that the health team needs to value the nurses’ spiritual care. The nurses’ mean Spirituality and Spiritual Care Rating Scale score is above the average level and varies by country. Spirituality and spiritual care perceptions are significantly higher among the nurses who work in Türkiye, are single, have a religious belief, embrace the Islamic faith, receive training for spiritual care, and use any spiritual practices to cope with difficulties or illnesses. 

To provide holistic nursing care and enhance the quality of patient care, it is recommended that nurses’ perceptions of spirituality and spiritual care and the effect of other factors that are likely to influence these perceptions should be evaluated at regular intervals. In addition, these assessments may contribute to detecting the barriers in front of nurses’ care practices, guiding administrators to remove these barriers. Moreover, instead of conducting spiritual care solely through religious officials or nurses, adopting a team approach and establishing spiritual support teams in health institutions worldwide is recommended. Finally, it would be beneficial to include spirituality and spiritual care in undergraduate nursing curricula and highlight the importance of spiritual care. 

## Figures and Tables

**Table 1:** Nurses’ sociodemographic characteristics and their perceptions of spirituality and spiritual care (n = 1090).

Characteristic	Türkiye (n = 319)	Albania (n = 429)	Italy (n = 342)	Total (n = 1090)	Test
	X ± SD (min–max)	X ± SD (min–max)	X ± SD (min–max)	X ± SD (min–max)	
**Mean age, years**	32.84 ± 7.41 (21–55)	36.36 ± 10.25 (20–65)	43.28 ± 11.52 (22–68)	37.50 ± 10.78 (20–68)	F = 95.455 ** p = 0.000
**Professional experience, years**	10.29 ± 8.07 (1–42)	11.40 ± 10.07 (1–42)	17.94 ± 11.75 (1–43)	13.13 ± 10.63 (1–43)	F = 57.607 ** p = 0.000
	**n/%**	**n/%**	**n/%**	**n/%**	
**Gender of nurses**					
Female	221 (69.3)	340 (79.3)	235 (68.7)	796 (73.0)	X = 13.951 * p = 0.001
Male	98 (30.7)	89 (20.7)	107 (31.3)	294 (27.0)	
**Marital Status**					
Single	191 (59.9)	126 (29.4)	77 (22.5)	394 (36.1)	X = 113.884 * p = 0.000
Married or have partner	128 (40.1)	303 (70.6)	265 (77.5)	696 (63.9)	
**Education**					
High school	16 (5.0)	7 (1.6)	66 (19.3)	89 (8.2)	X = 142.456 * p = 0.000
Bachelor’s degree	234 (73.4)	383 (89.3)	187 (54.7)	804 (73.8)	
Master or doctoral degree	69 (21.6)	39 (9.1)	89 (26.0)	197 (18.1)	
**Working Unit**					X = 211.777 * p = 0.000
Outpatient clinic	34 (10.7)	-	22 (6.4)	56 (5.1)	
Inpatient clinic	133 (41.7)	316 (73.7)	192 (56.1)	641 (58.8)	
Intensive care unit	73 (22.9)	76 (17.7)	17 (5.0)	166 (15.2)	
Emergency service	30 (9.4)	-	32 (9.4)	62 (5.7)	
Operating room	9 (2.8)	33 (7.7)	28 (8.2)	70 (6.4)	
Others	40 (12.5)	4 (0.9)	51 (14.9)	95 (8.8)	
**Job Role**					
Staff nurse	204 (63.9)	268 (85.8)	288 (84.2)	860 (78.9)	X = 126.387 * p = 0.000
Nurse manager	38 (11.9)	22 (5.1)	54 (15.8)	114 (10.5)	
Others	77 (24.2)	39 (9.1)	-	116 (10.6)	
**Choosing the profession willingly**					
Yes	217 (68.0)	419 (97.7)	320 (93.6)	956 (87.7)	X = 164,987 * p = 0.000
No	102 (32.0)	10 (2.3)	22 (6.4)	134 (12.3)	
**Job satisfaction**					
Yes	115 (36.1)	293 (68.3)	223 (65.2)	631 (57.9)	X = 116.652 * p = 0.000
Partially	162 (50.8)	131 (30.5)	109 (31.9)	402 (36.9)	
No	42 (13.2)	5 (1.2)	10 (2.9)	57 (5.2)	
**Having a religious belief**					
Yes	300 (94.0)	415 (96.7)	301 (88.0)	1016 (93.2)	X = 23.386 * p = 0.000
No	19 (6.0)	14 (3.3)	41 (12.0)	74 (6.8)	
**Type of religious belief**					
Islam	300 (94.0)	351 (81.8)	2 (0.6)	653 (59.9)	X = 765.653 * p = 0.000
Christianity	0	72 (16.8)	300 (87.7)	372 (34.1)	
Judaism	0	0	0	0	
Other	19 (6.0)	6 (1.4)	40 (11.7)	65 (6.0)	
**Regular religious practice**					
Yes	218 (68.3)	181 (42.2)	87 (25.4)	486 (44.6)	X = 124.588 * p = 0.000
No	101 (31.7)	248 (57.8)	255 (74.6)	604 (55.4)	
Type of religious practices ***					
To pray	33 (15.1)	19 (54.2)	35 (41.1)	87 (25.7)	X = 560.654 * p = 0.000
Meditation and salaat	87 (39.9)	2 (5.8)	2 (2.3)	91 (26.9)	
Mass ritual	5 (2.3)	14 (40.0)	37 (43.6)	56 (16.5)	
Reading the holy book	17 (7.8)	-	5 (5.9)	22 (6.5)	
Fasting	69 (31.6)	-	-	69 (20.5)	
To help (donation, charity, zakat, etc.)	7 (3.3)	-	6 (7.1)	13 (3.9)	
**Using** **spiritual care practices for the patients in your unit**					
Yes	75 (23.5)	6 (1.4)	66 (19.3)	147 (13.5)	X = 91.101 * p = 0.000
No	244 (76.5)	423 (98.6)	276 (80.7)	943 (86.5)	
Type of spiritual care practices ***					
Place of worship (chapel, mosque, etc.)	17 (22.7)	-	4 (6.3)	21 (14.5)	X = 230.581 * p = 0.000
Clergy visit	7 (9.4)	2 (33.3)	21 (32.8)	30 (20.8)	
Emotional support	41 (54.6)	3 (66.7)	5 (7.8)	49 (34.1)	
Collective prayer	1 (1.3)	-	34 (53.1)	35 (24.3)	
Moral support unit	9 (12.0)	-	-	9 (6.3)	
**Barriers to spiritual care practices**					
Nurses do not value spiritual care	32 (13.0)	30 (7.0)	25 (7.3)	87 (8.6)	X = 439.290 * p = 0.000
The health team does not value the nurses’ spiritual care	54 (22.0)	282 (65.7)	20 (5.8)	356 (35.0)	
Patients’ unwillingness to receive moral care support.	66 (26.8)	20 (4.7)	23 (6.8)	109 (10.7)	
Other	94 (38.2)	97 (22.6)	274 (80.1)	465 (45.7)	
**Believing that spiritual care practices are necessary**					
Yes	276 (86.5)	378 (88.1)	278 (81.3)	932 (85.5)	X = 7.528 * p = 0.023
No	43 (13.5)	51 (11.9)	64 (18.7)	158 (14.5)	
**Believing the knowledge of spiritual care practices is adequate**					
Yes	51 (16.0)	79 (18.4)	113 (33.0)	243 (22.3)	X = 493.657 * p = 0.000
Partially	203 (63.6)	313 (73.0)	0	516 (47.3)	
No	65 (20.4)	37 (8.6)	229 (67.0)	331 (30.4)	
**Receiving training for spiritual care**					
Yes	53 (16.6)	60 (14.0)	73 (21.3)	186 (17.1)	X = 7.346 * p = 0.025
No	266 (83.4)	369 (86.0)	269 (78.7)	904 (82.9)	
**Using any spiritual practices to cope with difficulties or illnesses**					
Yes	111 (34.8)	52 (12.1)	151 (44.2)	314 (28.8)	X = 103.085 * p = 0.000
No	208 (65.2)	377 (87.9)	191 (55.8)	776 (71.2)	
**Believing spiritual care practices have a healing effect**					
Yes	288 (90.3)	274 (63.9)	250 (73.1)	812 (74.5)	X = 67.689 * p = 0.000
No	31 (9.7)	155 (36.1)	92 (26.9)	278 (25.5)	

* Fisher’s exact test, ** one-way ANOVA, *** values are given as number (percentage) unless otherwise indicated; percentages are calculated using column totals. Multiple answers were given.

**Table 2:** Mean scores of spirituality and spiritual care rating scale items.

Items	Türkiye (n = 319) mean ± SD	Albania (n = 429) mean ± SD	Italy (n = 342) mean ± SD	Total (n = 1090) mean ± SD	Test
1. I think that nurses can provide spiritual care by inviting a religious official to the hospital on patient’s demand	3.41 ± 1.16	3.38 ± 1.00	3.39 ± 1.27	3.39 ± 1.14	F = 0.083 *p* = 0.920
2. I think that nurses can provide spiritual care by acting in a compassionate, concerned and positive manner while giving care	4.03 ± 1.07	3.30 ± 1.19	3.71 ± 1.30	3.64 ± 1.23	F = 35.118 *p* = 0.000
3. I think that spirituality is only concerned with a need to forgive and be forgiven	3.62 ± 0.98	2.54 ± 1.07	3.20 ± 1.27	3.06 ± 1.20	F = 89.041 *p* = 0.000
4. I think that spirituality involves only going to a place of worship (mosque/church)	4.27 ± 0.78	2.99 ± 1.52	3.93 ± 0.99	3.66 ± 1.30	F = 120.012 *p* = 0.000
5. I think that spirituality is not concerned with belief in God or a supreme power and worship	2.79 ± 1.17	2.30 ± 0.87	2.89 ± 1.21	2.63 ± 1.11	F = 33.465 *p* = 0.000
6. I think that spirituality is concerned with finding meaning in the good and bad events of our lives	3.48 ± 0.96	3.01 ± 1.041	3.53 ± 1.28	3.31 ± 1.12	F = 26.270 *p* = 0.000
7. I think that nurses can provide spiritual care by allocating time for patients to support them in time of need	3.47 ± 1.05	3.19 ± 1.16	3.34 ± 1.24	3.32 ± 1.16	F = 5.467 *p* = 0.004
8. I think that nurses can provide spiritual care by helping patients in finding the meaning and causes of their illnesses	3.57 ± 0.94	3.16 ± 1.12	3.53 ± 1.23	3.40 ± 1.13	F = 16.400 *p* = 0.000
9. I think that spirituality is concerned with having hope for life	3.70 ± 0.85	3.17 ± 1.06	3.34 ± 1.22	3.38 ± 1.08	F = 23.471 *p* = 0.000
10. I think that spirituality is about living one’s life “here and now”	3.40 ± 0.91	2.89 ± 1.13	3.59 ± 1.28	3.26 ± 1.16	F = 40.613 *p* = 0.000
11. I think that nurses can provide spiritual care by giving patients enough time to explain and discuss their fears, worries and sorrows, and listening to them	3.71 ± 0.91	3.12 ± 1.15	3.55 ± 1.28	3.43 ± 1.16	F = 27.971 *p* = 0.000
12. I think that spirituality is a unifying force which enables one to be at peace with oneself and his or her environment	4.00 ± 0.72	3.11 ± 1.16	2.51 ± 1.19	3.18 ± 1.21	F = 163.995 *p* = 0.000
13. I think that spirituality does not involve areas such as art, creativity and self-expression	3.52 ± 1.03	2.65 ± 0.77	2.37 ± 1.35	2.82 ± 1.16	F = 106.914 *p* = 0.000
14. I think that nurses can provide spiritual care by showing respect for the privacy, dignity, religion and cultural beliefs of a patient	4.07 ± 0.83	3.17 ± 1.18	3.10 ± 1.21	3.41 ± 1.18	F = 80.134 *p* = 0.000
15. I think that spirituality involves personal friendships and relationships	3.62 ± 0.89	2.26 ± 0.83	2.21 ± 1.05	2.64 ± 1.11	F = 251.389 *p* = 0.000
16. I think that spirituality does not apply to those who do not have a belief in God/Supreme Power	3.73 ± 1.04	3.09 ± 1.44	2.74 ± 1.25	3.17 ± 1.33	F = 50.810 *p* = 0.000
17. I think that spirituality is a concept that includes morality	3.97 ± 0.91	2.25 ± 0.79	2.11 ± 0.56	2.71 ± 1.11	F = 613.255 *p* = 0.000
**Total Score**	3.66 ± 0.43	2.91 ± 0.58	3.11 ± 0.58	3.20 ± 0.62	F = 180.060 *p* = 0.000

**Table 3:** Spirituality and Spiritual Care Rating Scale (SSCRS) scores and selected independent variables.

Characteristic	SSCR Scale	
	Mean ± SD	Test
**Country**		
Türkiye _(a)_	3.66 ± 0.43	F = 180.060 * p = 0.000 Post hoc Tukey test a > c > b *p* = 0.000
Albania _(b)_	2.91 ± 0.58	
Italy _(c)_	3.11 ± 0.58	
**Age**		
20–30 years old _(a)_	3.26 ± 0.62	F = 16.768 *p* = 0.000 Post hoc Tukey test a > b > c *p* = 0.000
31–45 _(b)_	3.25 ± 0.60	
≥45 years old _(c)_	3.01 ± 0.63	
**Gender**		
Female	3.17 ± 0.62	t = −1.918 ** p = 0.055
Male	3.25 ± 0.61	
**Marital Status**		
Single	3.28 ± 0.66	t = 3.339 *p* = 0.001
Married or have partner	3.14 ± 0.60	
**Education**		
High school _(a)_	3.05 ± 0.65	F = 3.646 *p* = 0.026 Post hoc Tukey test c > a *p* = 0.019
University _(b)_	3.19 ± 0.60	
Postgraduate _(c)_	3.27 ± 0.70	
**Working Unit**		
Outpatient clinic _(a)_	3.51 ± 0.51	F = 7.041 *p* = 0.000 Post hoc Tukey test a > b *p* = 0.000 a > e *p* = 0.002 c > a *p* = 0.002 a > e *p* = 0.002
Inpatient clinic _(b)_	3.13 ± 0.63	
Intensive care unit _(c)_	3.33 ± 0.55	
Emergency service _(d)_	3.27 ± 0.61	
Operating room _(e)_	3.09 ± 0.59	
Other _(f)_	3.27 ± 0.70	
**Professional experience**		
<5 years _(a)_	3.24 ± 0.64	F = 3.980 *p* = 0.019 Post hoc Tukey test a > c *p* = 0.033
6–15 years _(b)_	3.23 ± 0.57	
>15 years _(c)_	3.12 ± 0.65	
**Having a religious belief**		
Yes	3.21 ± 0.62	t = 2.656 *p* = 0.008
No	3.01 ± 0.64	
**Type of religious belief**		
Islam _(a)_	3.28 ± 0.60	F = 14.959 *p* = 0.000 Post hoc Tukey test a > b *p* = 0.000
Christianity _(b)_	3.06 ± 0.64	
Other _(c)_	3.17 ± 0.63	
**Regular religious practice**		
Yes	3.29 ± 0.70	t = 4.529 *p* = 0.000
No	3.12 ± 0.54	
**Receiving training for spiritual care**		
Yes	3.09 ± 0.82	t = −2.546 *p* = 0.011
No	3.22 ± 0.57	
**Using any spiritual practices to cope with difficulties or illnesses**		
Yes	3.33 ± 0.70	t = 4.559 *p* = 0.000
No	3.14 ± 0.58	
**Believing spiritual care practices have a healing effect**		
Yes	3.26 ± 0.66	t = 5.821 *p* = 0.00
No	3.01 ± 0.45	

* One-way ANOVA, ** independent *t*-test. Statistically significant *p* values are indicated in bold (*p* < 0.05); SD: standard deviation.

**Table 4:** Multiple linear regression analysis of factors potentially influential on the SSCRS scores.

Factors	Beta	t-Statistics	*p*
Country (Türkiye)	−0.427	−11.147	0.000
Age (20–30 years old)	−0.090	−1.827	0.068
Marital status (single)	0.073	2.381	0.017
Education (postgraduate)	0.020	0.694	0.488
Working unit (outpatient clinic)	0.032	1.155	0.248
Professional experience (<5 years)	0.037	0.785	0.433
Having a religious belief	−0.109	−3.263	0.001
Type of religious belief (Islam)	0.160	4.097	0.000
Regular religious practice	0.013	0.403	0.687
Receiving training for spiritual care	0.108	3.726	0.000
Using any spiritual practices to cope with difficulties or illnesses	−0.178	−5.894	0.000

R^2^ = 0.171; F = 22.106; *p* = 0.000; Durbin–Watson = 1.637.

## Data Availability

The data that support the findings of this study are available from the corresponding author, H.D., upon reasonable request.
